# A Case of Traumatic Tetanus Cured by the Removal of a Piece of Glass

**Published:** 1854-11

**Authors:** C. Hixson

**Affiliations:** Harrisonville, Ind.


### MEDICINE AND SURGERY.

NOVEMBER, 1854. NEW SERIES. VOL. II.—NO. 11.

### ART. I.—A CASE OF TRAUMATIC TETANUS CURED BY THE REMOVAL OF A PIECE OF GLASS, WHICH HAD BEEN IMBEDDED IN THE ARCH OF THE FOOT FOR TWO YEARS AND SEVEN MONTHS.

BY DR. C. HIXSON, OF HARRISONVILLE, IND.

ON the 8th of May, 1853, I was called to see Mrs. W——, who was laboring under tetanic convulsions of most alarming severity. The jaws were firmly set, the muscles of the extremities rigid, and the dorsal muscles in a most extreme degree of constriction, alternating with complete relaxation. Upon inquiry I found that she had been in this condition for about ten hours, but had been the subject of slight convulsions for several days, previous. My attention was directed to her right foot, which I found to be enormously swollen, and on questioning her husband, learned, that a little over two years and a half before, while bare-footed, she had trodden upon a piece of window-glass, which entered the outside of the arch of the foot, near the cuneo-cuboid articulation, taking a direction upward and inward. A portion of glass was removed at the time of the accident, which was supposed to be all, as the wound readily healed.

Nothing more was thought of the matter, as she suffered no annoyance from it, till some months after her removal to this state. Being a woman of stout constitution, she wore no shoes during a great portion of her time, but upon wearing, for a few days, a pair of shoes smaller than ordinary, she began to complain of a pricking sensation in the sole of the foot, which continued to increase in severity until she was brought to the condition which I have previously described.

I immediately administered chloroform to the extent of producing complete insensibility, and proceeded to a minute examination of the foot. My diagnosis was, that there remained a spiculum of glass in the foot, which had become encysted, and which, upon the occasion of the foot being compressed by a shoe too small for it, had ruptured the sack in which it was enclosed, and had now produced the irritation which I witnessed. I determined at once to make an incision into the arch of the foot, and ascertain if such were not the case. I accordingly made an incision, commencing a little below and forward of the articulation of the calcaneum and astragalus, which I continued for about an inch and a half in depth, when the knife struck some hard substance. I then with a small pair of forceps removed a piece of glass measuring an inch and a quarter in length by half an inch in width at the base.

Nothing afforded me greater satisfaction than the feeling of comfort which my patient expressed upon her return to consciousness, after the removal of the foreign body, thus verifying the correctness of my diagnosis, that the tetanic condition was produced and kept up by a foreign body remaining imbedded in the foot. With a probe I now ascertained that there was nothing more remaining to keep up the irritation, the spasms ceased, my patient's system became completely relaxed, and after injecting a small quantity of tincture of opium into the incision and exhibiting a half grain of sulph. morphia, I left her comfortable. The spasms have never returned, the swelling soon disappeared, and she has ever since enjoyed excellent health.

This case is particularly remarkable for the fact that a foreign body shaped like this, should remain so long (two years and seven months nearly) in a part organized like the foot, without creating irritation, or working its way out.

Had the foot not been unusually compressed, it might have remained there during life, without ever having been known, even to the woman herself.

SEPT. 1854.

